# Crowdsourcing Medical School Admissions Data: Development and Analysis of the CycleTrack Platform

**DOI:** 10.2196/83087

**Published:** 2026-05-22

**Authors:** Daniel Boris Amusin, Isabelle Hua, Arthi Shankar Kozhumam

**Affiliations:** 1 Medical Scientist Training Program, Feinberg School of Medicine, Northwestern University, 303 E Chicago Ave, Morton 1-670, Chicago, IL, 60611, United States, 1 312-503-5232; 2 Medical Scientist Training Program, School of Medicine, University of Colorado, Aurora, CO, United States

**Keywords:** undergraduate medical education, medical school admission, data accessibility, crowdsourcing, CycleTrack

## Abstract

Using a crowdsource model, we developed CycleTrack, a web-based platform that tracks and aggregates medical school application data in real-time. From May 2022 to January 2026, 34,763 users registered on the platform, of whom 20,354 tracked a cumulative 415,837 doctor of medicine (MD), MD–doctor of philosophy (PhD), doctor of osteopathic medicine (DO), and DO-PhD applications. The volume of applications tracked per program strongly correlated with the total number of applications reported in official data from the Association of American Medical Colleges and American Association of Colleges of Osteopathic Medicine (Spearman ρ≥0.91; *P*<.001); however, statistically significant differences in demographics, grade point average, and Medical College Admission Test (MCAT) scores were observed between CycleTrack users and national averages. Despite this, the CycleTrack database accurately captured the cadence of interview invitations from the Northwestern University and University of Michigan MD programs. Aggregated data from the CycleTrack database offered a glimpse into application cycle dynamics, such as demonstrating the relatively longer DO interview cycle compared to MD and MD-PhD programs. Use data from CycleTrack suggest demand for open and transparent information about statistics and timing of admissions decisions among medical school applicants.

## Introduction

Medical school admission in the United States is highly competitive, with matriculation rates below 45% [1,2]. Applicants frequently seek to optimize acceptance chances by consulting online resources. While select data are available through the Medical School Admissions Requirements and Choose DO Explorer tools, there are remaining information gaps in the timing of rolling admissions decisions and data specific to dual-degree applicants. Applicants have frequently turned to online forums such as the Student Doctor Network or Reddit to share and discuss this information [3]. However, anecdotal experiences from individual users may not reflect empirical evidence and mislead applicants, which may especially discourage low-income and minority background applicants [4,5].

In the absence of official data, crowdsourcing information may support applicants. Tools such as the American Medical College Application Service (AMCAS) Tracker [6] visualize the timing of application verification, and MDApplicants [7] displays individual applicant profiles. These tools lack key information such as the timing of interview invitations and admissions decisions. An early attempt to aggregate interview invitation dates by the Student Doctor Network user “TheDataKing” relied on end-of-cycle surveys that required entry into a Google Form [8]. However, the lack of timeliness and the need for manual entry, curation, and visualization limited uptake.

Empowering applicants requires novel methods for increasing data availability. Here, we describe CycleTrack, a novel platform that bridges the individual application tracking spreadsheet with transparent automated data aggregation.

## Methods

### Overview

CycleTrack is a free medical school admissions tracking platform built using the Python Flask framework [9,10]. Applicants register on the website, create school lists, share optional demographic/statistical information, and enter dates of actions such as interview invitations or acceptances. Data are collected using a standardized interface from email-verified users and stored in a SQLite3 database. Input validation ensures that data fit AMCAS demographic categories, grade point average and Medical College Admission Test (MCAT) scores are within appropriate bounds, and dates are between May of the application year and August of the matriculation year. The database is queried hourly to generate publicly visible summary statistics/graphs on school-specific data explorer pages. To maintain privacy, summary statistics are only reported when at least 5 points are available, and dates of actions are never linked to other user information.

To characterize CycleTrack users, demographics, and scores, applications from the platform were compared to national data provided by the Association of American Medical Colleges (AAMC) and American Association of Colleges of Osteopathic Medicine (AACOM) [1,2].

Tracking the cadence of interview invitations is a common use of CycleTrack, and we explored the validity and utility of self-reported data for this purpose. Official dates of interview invitations from Northwestern University and the University of Michigan were compared to those of CycleTrack users. Additionally, the proportions of interview invitation entries in CycleTrack from completed application cycles (2022‐2025) were plotted over time to examine differences in the dissemination of interviews between years and program types (total: N=24,941; doctor of medicine [MD]: n=18,465; doctor of osteopathic medicine [DO]: n=3698; MD–doctor of philosophy [PhD]: n=2778). All statistics and graphs were generated using R v4.5.0 (The R Foundation for Statistical Computing) and ggplot2 [11].

### Ethical Considerations

Our study was confirmed to be nonhuman subjects research by the Northwestern University Institutional Review Board (#STU00225793).

## Results

### Applications and Users

**Figure 1.**
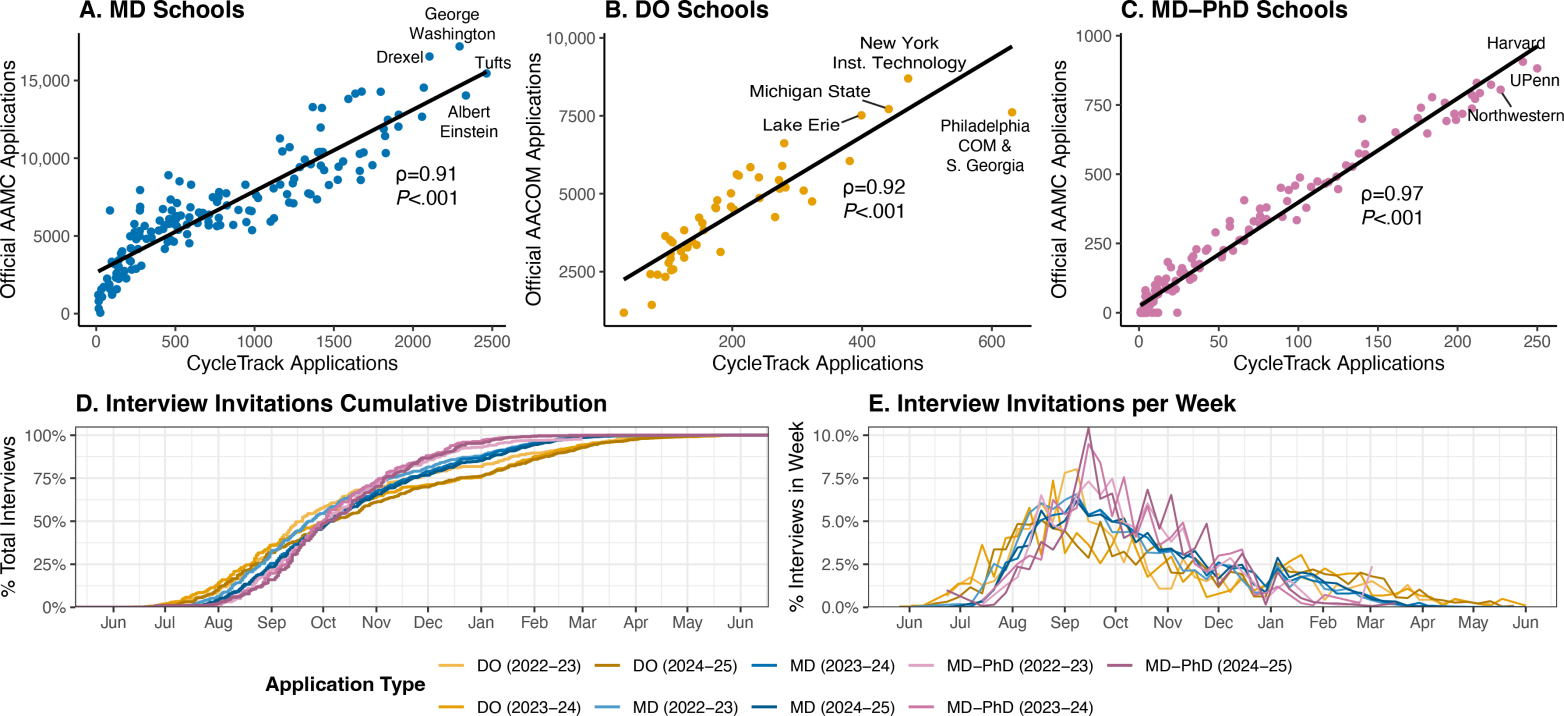
Commonly tracked programs and utility of CycleTrack in examining the interview cycle. (A-C) The most commonly tracked programs in CycleTrack are associated with the number of applications submitted to programs reported by the Association of American Medical Colleges (AAMC) and American Association of Colleges of Osteopathic Medicine (AACOM) during the 2024‐2025 cycle (Spearman ρ≥0.91; *P*<.001) [1,2]. (D) Cumulative distributions of interview invitations stratified by doctor of medicine (MD), doctor of osteopathic medicine (DO), and MD–doctor of philosophy (PhD) applicants for the 2022‐2023, 2023‐2024, and 2024‐2025 application cycles. Cumulative distributions were significantly different (9-sample Anderson-Darling *P*<.001). (E) The weekly percent of total interviews sent for each program type and year. Individual plots for each program type are shown and discussed in Multimedia Appendix 1*.* DO-PhD applications were excluded from temporal modeling due to an insufficient sample size*.*

From May 2022 to January 2026, 34,763 email-verified users registered on the platform. Of these, 20,354 tracked at least one application (MD: n=18,848; MD-PhD: n=1592; DO: n=4456; DO-PhD: n=67), submitting a total of 415,837 applications (MD: n=359,440; MD-PhD: n=26,018; DO: n=30,217; DO-PhD: n=162). Google Analytics data of website visitors throughout the 2024-2025 and 2025-2026 cycles identified over 257,000 unique visitors, with the most traffic to the data explorer. The number of tracked applications strongly correlated with the total applications for each school reported by the AAMC and AACOM (Figure 1A-C), and in the 2024‐2025 cycle, CycleTrack applications represented a median 13.5% (range 1.1%‐44.1%) of total submitted applications per program (MD: 10.0%, 1.1%‐44.1%; DO: 4.1%, 2.7%‐8.3%; MD-PhD: 24.4%, 4.9%‐29.6%).

Of users who tracked applications, 80% reported demographic information. Demographics reported in the 2024‐2025 application cycle are summarized in Table 1. CycleTrack users were more likely to be men, be White or Asian, and score higher compared to national statistics.

**Table 1.** Comparison of CycleTrack user demographics from the 2024‐2025 application cycle to national data reported by the Association of American Medical Colleges (AAMC) and American Association of Colleges of Osteopathic Medicine (AACOM) [1,2].

	CycleTrack^a^	AAMC (MD^b^/MD-PhD^c^) [1] or AACOM (DO^d^) [2]	*P* value^e^
MD			
Gender, n (%)			<.001
Man	2321 (45.4)	23,072 (42.2)	
Woman	2731 (53.4)	31,292 (57.2)	
Other, transgender, nonbinary	60 (1.2)	265 (0.5)	
Race/ethnicity, n (%)			<.001
Asian	1682 (34.0)	14,585 (26.7)	
Black	265 (5.4)	5022 (9.2)	
Hispanic	383 (7.8)	3752 (6.9)	
White	2180 (44.1)	18,597 (34.0)	
Other, multiple	430 (8.7)	12,743 (23.3)	
Age at matriculation (years), mean (SD)	23.9 (2.6)	NR^f^	—^g^
GPA^h^, mean (SD)	3.79 (0.21)	3.67 (0.33)	<.001
MCAT^i^, mean (SD)	513.4 (6.5)	506.3 (10.0)	<.001
MD-PhD			
Gender, n (%)			ND^j^^,^^k^
Man	179 (48.2)	NR	
Woman	182 (49.1)	NR	
Other, transgender, nonbinary	10 (2.7)	NR	
Race/ethnicity, n (%)			<.001
Asian	139 (39.0)	619 (30.3)	
Black	23 (6.5)	210 (10.3)	
Hispanic	13 (3.7)	116 (5.7)	
White	145 (40.7)	619 (30.3)	
Other, multiple	36 (10.1)	476 (23.3)	
Age at matriculation (years), mean (SD)	23.7 (2.0)	NR	—
GPA, mean (SD)	3.83 (0.22)	3.71 (0.33)	<.001
MCAT, mean (SD)	516.2 (6.6)	510.4 (10.4)	<.001
DO			
Gender, n (%)			ND^l^
Man	586 (43.2)	9464 (40.9)^l^	
Woman	757 (55.8)	13,602 (58.8)^l^	
Other, transgender, nonbinary	14 (1.0)	48 (0.2)^l^	
Race/ethnicity, n (%)			<.001
Asian	399 (30.5)	6331 (27.4)	
Black	79 (6.0)	1844 (8.0)	
Hispanic	107 (8.2)	2583 (11.2)	
White	618 (47.2)	9905 (42.9)	
Other, multiple	106 (8.1)	2451 (10.6)	
Age at matriculation (years), mean (SD)	24.5 (3.1)	NR	—
GPA, mean (SD)	3.70 (0.25)	3.58 (NR^m^)	<.001
MCAT, mean (SD)	508.2 (6.3)	502.4 (NR^m^)	<.001

a As entry of demographic information is optional for CycleTrack users, not all variables have the same number of total responses. Users who applied to multiple program types are counted for each program type they applied to. Therefore, these analyses are not statistically independent of one another.

b MD: doctor of medicine.

c PhD: doctor of philosophy.

d DO: doctor of osteopathic medicine.

e Categorical data were compared by *χ*2 test, GPA was compared by Wilcoxon signed rank test, and MCAT was compared by 1-sample t test.

f NR: not reported; data not publicly disclosed by the AAMC or AACOM.

g Not applicable.

h GPA: grade point average.

i MCAT: Medical College Admission Test.

j ND: not done; statistical tests that were not performed.

k The AAMC does not publicly report the gender distribution of MD-PhD applicants. The gender distribution of applications to MD-PhD programs is 49.3% men, 49.1% women, and 1.6% other or declined to report [1]. As the number of applications submitted per applicant may vary by gender, these percentages may not fully reflect the distribution of applicants.

l The American Association of Colleges of Osteopathic Medicine Application Service (AACOMAS) reports gender using binary categories (male, female, not disclosed). The American Medical College Application Service (AMCAS) uses identity-based categories (man, woman, nonbinary, etc). As CycleTrack uses categories defined by AMCAS, no direct comparison to AACOMAS data was performed.

m The most recent AACOMAS applicant GPA and MCAT information at the time of writing are reported for the 2023‐2024 application cycle.

### Interview Invitations

Compared to official data from the University of Michigan and Northwestern University MD programs, CycleTrack accurately captured 79% to 96% of the weeks on which interview invitations were released (Multimedia Appendix 2). Modeling the cumulative distribution of interviews across three application cycles demonstrated that DO interview invitations exhibited the greatest yearly fluctuation and that DO and MD-PhD interview cycles are longer and shorter than MD, respectively (Figure 1D-E, further discussed in Multimedia Appendix 1).

## Discussion

CycleTrack extends prior tools by serving as the first website to centralize real-time application tracking and distribution of aggregated statistics. The volume of users/traffic demonstrates demand for free, publicly accessible tools and information about application timelines. Following the launch of CycleTrack, other online platforms (eg, Admit) have implemented and iterated on its model for both medical school and residency admissions.

CycleTrack demographics deviated from AAMC/AACOM statistics, which may influence the accuracy of the explorer tool. While hesitancy to report data among lower-scoring or underrepresented students may be a contributing factor, it is also likely that online word-of-mouth advertising via forums used by CycleTrack is insufficient to reach some populations of applicants. Furthermore, while our analysis of reported interview dates provides utility for understanding the overall cadence of the interview cycle, as survival analysis techniques were not implemented, the chronological decline in reported invitations may reflect survival bias and user abandonment rather than true institutional behavior. Greater advertisement and uptake of such aggregation platforms may address several of these limitations. However, data transparency at the institutional and national levels is feasible as demonstrated by AAMC/AACOM tools and the University of Michigan #UMichMedMondays program. We urge medical schools and their national bodies to adopt clear and transparent models for sharing real-time application metrics so that platforms such as CycleTrack are obsolete.

Despite its limitations, CycleTrack has advanced data aggregation efforts among premedical applicants. It has uniquely captured temporal trends of admissions actions, provided dual-degree–specific data, and built a database for data-driven inquiries into applicant questions. Furthermore, it demonstrates the power of a student initiative to improve medical school applications for all.

## Supplementary material

10.2196/83087 Multimedia Appendix 1 Interview invitations by program type and application cycle.

10.2196/83087 Multimedia Appendix 2 External validation of CycleTrack interview data.

## Abbreviations

AACOM: American Association of Colleges of Osteopathic Medicine
AAMC: Association of American Medical Colleges
AMCAS: American Medical College Application Service
DO: doctor of osteopathic medicine
MCAT: Medical College Admission Test
MD: doctor of medicine
PhD: doctor of philosophy
