# Supplementary material for: Crowdsourcing Medical School Admissions Data: Development and Analysis of the CycleTrack Platform
Source: J Med Internet Res. 2026 May 22;28:e83087. doi: 10.2196/83087 (PMC13197106; doi:10.2196/83087)
Supplement: Multimedia Appendix 1 [file jmir-v28-e83087-s001.pdf]

Multimedia Appendix 1. Interview invitations by program type and application cycle

Medical school interview invitations are typically sent on a rolling basis. Therefore, the cadence by which programs send invitations and the relative quantity of invitations remaining are of particular interest to applicants. We used the CycleTrack database to model the cumulative distribution of interviews for MD, DO, and MD-PhD programs for the 2022-2023 (MD: n=3208; DO: n=460; MD-PhD: n=629), 2023-2024 (MD: n=5392; DO: n=1010; MD-PhD: n=833), and 2024-2025 (MD: n=9865; DO: 2228; MD-PhD: 1316) application cycles and assessed differences between cycles using a three sample Anderson-Darling test (Figure A1). DO-PhD applications were excluded due to insufficient sample size. For each program type, all three application cycle cumulative distributions were significantly different ($P<.05$). However, for MD and MD-PhD, the effect size was small with differences of up to ~2 weeks. For all three program types, distributions demonstrated that interview invitations began and ended at relatively similar time points (MD: mid-July to early March, DO: late June to late April, MD-PhD: early August to early February). The DO interview cycles exhibited the most variability with the 2022-2023 cycle shifted earlier compared to the other two. However, this may be partially influenced by a relatively smaller sample size compared to the other two years.

Figure A1. The cumulative distribution of interview offers (A-C) and weekly percent of total interview invitations (D-F) for MD, DO, and MD-PhD programs for 2022-2023, 2023-2024, and 2024-2025 application cycles.

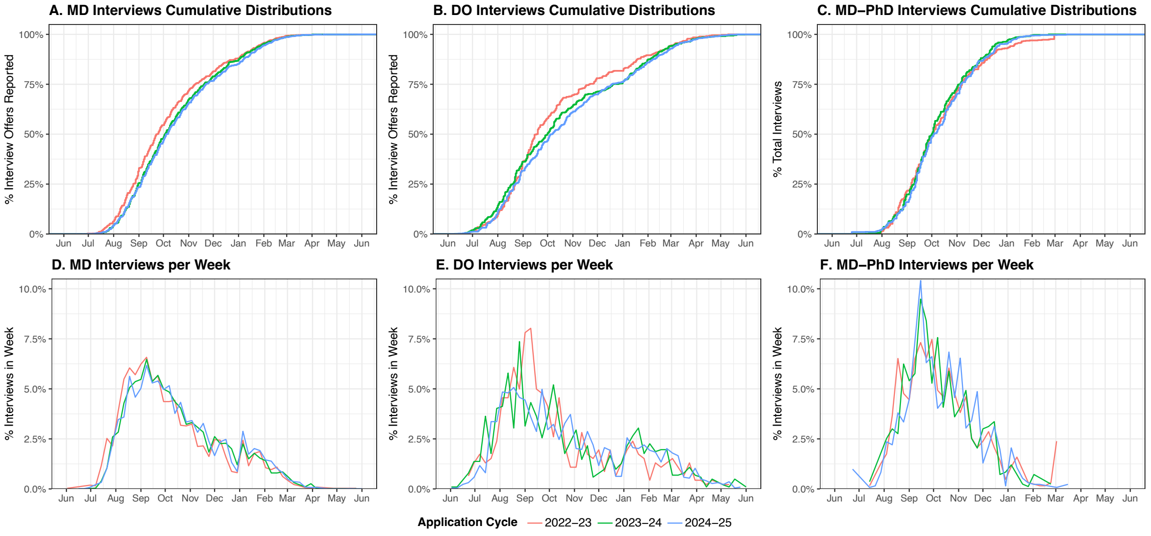

Rolling admissions advantage applicants who submit early as they can maximize the number of interview pools for which they are considered. However, this must be balanced with sufficient time to write compelling statements. Therefore, we next examined the quartiles of the earliest interview invitation dates tracked by CycleTrack users for each program using the two application cycles with the greatest amount of data, 2023-2024 and 2024-2025 (Table A1). These data further reinforced the trend that DO programs send interview invitations earlier while MD-PhD send them later and identified that the majority of programs have begun sending interview invitations by September.

Table A1. Quartile dates by which individual programs have sent their first interview invitation.

Quartile	MD		DO		MD-PhD	
	2023-2024	2024-2025	2023-2024	2024-2025	2023-2024	2024-2025
1st	8/7	8/1	7/16	7/18	8/14	8/5
2nd	8/16	8/14	8/8	8/1	9/7	9/4
3rd	8/31	8/29	8/22	8/23	10/4	9/23
