# Supplementary material for: Crowdsourcing Medical School Admissions Data: Development and Analysis of the CycleTrack Platform
Source: J Med Internet Res. 2026 May 22;28:e83087. doi: 10.2196/83087 (PMC13197106; doi:10.2196/83087)
Supplement: Multimedia Appendix 2 [file jmir-v28-e83087-s002.pdf]

Multimedia Appendix 2. External validation of CycleTrack interview data

To examine whether CycleTrack data accurately reflects the cadence of interview invitations throughout the medical school admissions cycle, user-reported data from the 2024-2025 and 2025-2026 (through Jan 31, 2026) admissions cycles was compared to official weeks of interview invitations sent by the Northwestern University Feinberg School of Medicine and the University of Michigan Medical School. Data from Northwestern University were provided by the Office of Admissions and data from the University of Michigan were collected from public #UMichMedMonday(s) hashtag posts on the X social media platform (https://x.com/UMichMedSchool). These public interview invitation dates were for the MD program only, as the University of Michigan's MD-PhD program sent out their invitations separately.

The proportion of weeks in which interview invitations were sent and tracked by CycleTrack users are shown in Figure A2. CycleTrack reflected 96.2%, 84.2%, and 63.6% of weeks in which invitations were sent by the Northwestern University MD (2024-2025: 27 of 28, 2025-2026: 24 of 25), University of Michigan MD (2024-2025: 17 of 19, 2025-2026: 15 of 19), and Northwestern MD-PhD (2024-2025: 6 of 10, 2025-2026: 8 of 12) programs respectively. Importantly, the weeks missing from CycleTrack for the Michigan MD and Northwestern MD-PhD programs trended with fewer total interview offers sent by the program and reached statistical significance in three of the four evaluated cohorts (Table A2). These data suggest that CycleTrack likely captures major trends in interview invitations, but may miss smaller batches of invitations, particularly for programs with smaller numbers of total seats such as the Northwestern University MD-PhD program. However, it is important to note that this statistical analysis is likely to be underpowered due to the small sample size of weeks not represented in CycleTrack and should be interpreted with caution. Furthermore, this external validation was performed against a convenience sample of two highly resourced Midwestern universities, and these data may not generalize to the diverse spectrum of medical schools within the United States.

Figure A2. The proportion of weeks of true interview invitations represented in the CycleTrack database for three programs across two application cycles are shown.

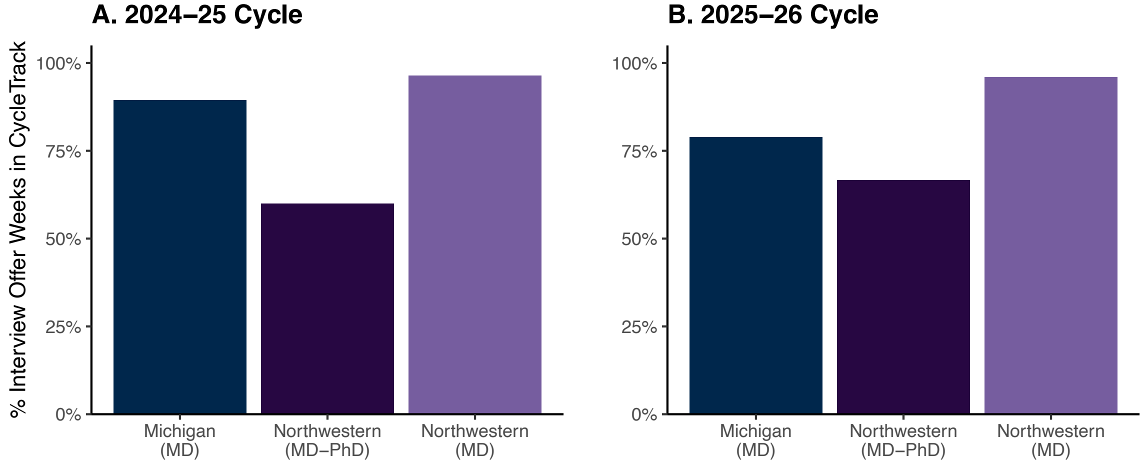

Table A2. Comparison of the mean (standard deviation) number of interviews sent by the University of Michigan Medical School MD program and Northwestern University MD-PhD program, stratified by weeks represented or not represented in CycleTrack.

Program, Cycle	Weeks Represented in CycleTrack, Mean Total Interview Invitations (SD)	Weeks Not Represented in CycleTrack, Mean Total Interview Invitations (SD)	*P* value[a]
Michigan MD, 2024-2025	21.2 (9.3)	13 (1.4)	0.08
Michigan MD, 2025-2026	21.6 (8.2)	9.5 (4.1)	<0.01
Northwestern MD-PhD, 2024-2025	19.5 (6.7)	1.25 (0.5)	<0.01
Northwestern MD-PhD, 2025-2026	14.5 (9.4)	1 (0)	<0.01

[a]Statistical differences were calculated using an Exact Wilcoxon Rank Sum test.
